# A Cotton Cyclin-Dependent Kinase E Confers Resistance to *Verticillium dahliae* Mediated by Jasmonate-Responsive Pathway

**DOI:** 10.3389/fpls.2018.00642

**Published:** 2018-05-24

**Authors:** Xiancai Li, Yakun Pei, Yun Sun, Nana Liu, Ping Wang, Di Liu, Xiaoyang Ge, Fuguang Li, Yuxia Hou

**Affiliations:** ^1^College of Science, China Agricultural University, Beijing, China; ^2^State Key Laboratory of Cotton Biology, Institute of Cotton Research, Chinese Academy of Agricultural Sciences, Anyang, China

**Keywords:** cotton (*Gossypium hirsutum*), GhCDKE, *Verticillium dahliae*, jasmonate signal, VIGS

## Abstract

Many subunits of the Mediator transcriptional co-activator complex are multifunctional proteins that regulate plant immunity in *Arabidopsis*. Cotton cyclin-dependent kinase E (GhCDKE), which is a subunit of the cotton (*Gossypium hirsutum*) Mediator complex, has been annotated, but the biological functions of this gene associated with regulating disease resistance have not been characterized. Here, we cloned *GhCDKE* from cotton and confirmed that GhCDKE belonged to the E-type CDK family in the phylogenetic tree, and, as in other eukaryotes, we found that GhCDKE interacted with C-type cyclin (GhCycC) by yeast two-hybrid and luciferase complementation imaging assays. Expression of *GhCDKE* in cotton was induced by *Verticillium dahliae* infection and MeJA treatment, and silencing of *GhCDKE* expression in cotton led to enhanced susceptibility to *V. dahliae*, while overexpression of *GhCDKE* in *Arabidopsis thaliana* enhanced resistance to this pathogen. Transgenic expression assay demonstrated that the transcriptional activity of *GhPDF1.2_pro_:LUC* in *GhCDKE*-silenced cotton was dramatically inhibited. In addition, the expression of jasmonic acid (JA)-regulated pathogen-responsive genes was dramatically upregulated in *GhCDKE*-overexpressed plants after inoculation with *V. dahliae*, and the roots of *GhCDKE*-overexpressed *A. thaliana* were more susceptible to JA treatment. These results indicated that GhCDKE regulates resistance against *V. dahliae* and that this resistance is mediated by JA response pathway.

## Introduction

Cotton is an important crop globally because of its fiber. However, yield and quality can be negatively affected by the fungus *Verticillium dahliae* (Fradin and Thomma, 2006). Several factors make it difficult to control this disease. First, the host range of *Verticillium dahliae* is broad, and the fungus can survive in the soil for many years, even in the absence of hosts (Ma et al., 1997). Moreover, studies have shown that pathogenic types of *V. dahliae* in cotton fields comprise a highly diverse community, which continuously changes from weak to strong type (Zhu et al., 2004).

Although this disease is particularly difficult to control, efforts have been made to investigate the molecular mechanisms underlying cotton resistance to *V. dahliae* infection. Many resistance and resistance-related genes have been discovered. For example, the immune receptor Ve1, which is responsible for *V. dahliae* resistance, has been cloned from tomato (Kawchuk et al., 2001; de Jonge et al., 2012). Importantly, it has been shown that interfamily transfer of *Ve1* into *Arabidopsis thaliana* confers resistance to race 1 strains of *V. dahliae* (Fradin et al., 2011). Using a virus-induced gene silencing (VIGS) approach, the signaling cascade downstream of Ve1 requires both EDS1 and NDR1. ACIF, MEK2, BAK1, and NRC1 also act as positive regulators of *Ve1* (Fradin et al., 2009). In addition, sets of genes/proteins, including components in ethylene (ET)-, salicylic acid (SA)-, and jasmonic acid (JA)-mediated signaling pathways, have been shown to be functionally related to *V. dahliae* resistance (Xu et al., 2014). These studies have taken important steps toward discovering the complex disease resistance mechanisms against *V. dahliae* infection in cotton (Kawchuk et al., 2001; Fradin et al., 2009; Xu et al., 2014).

Transcription regulation of defense-related genes is important in plant–pathogen interactions. In eukaryotes, a highly conserved multiprotein complex called the Mediator complex mediates the interaction between transcription regulators and RNA polymerase II (RNAPII) (Kim et al., 1994; Koleske and Young, 1994). The core of the Mediator is composed of three discrete modules (head, middle, and tail) and a separable kinase module consisting of Mediator complex subunit 12 (MED12), MED13, cyclin-dependent kinase 8 (CDK8), and C-type cyclin (CycC; Guglielmi et al., 2004; Andrau et al., 2006). Different Mediator subunits interact with particular transcription activators to transfer various signals to the RNAPII transcription complex and results in the transcription of specific pathway genes (Donner et al., 2007). For example, the subunit MED2/MED32 regulates the expression of cold-responsive genes in *Arabidopsis* (Hemsley et al., 2014).

Recent studies have shown that Mediator subunits are associated with plant immunity to bacterial and fungal infection. For instance, MED25 is a positive regulator of JA-responsive gene expression, and mutation of this gene leads to increased resistance to the hemibiotroph *Fusarium oxysporum* and susceptibility to the necrotrophic pathogens *Botrytis cinerea* and *Alternaria brassicicola* (Kidd et al., 2009; Çevik et al., 2012; Chen et al., 2012). MED15/NRB4 functions downstream of NPR1 to regulate the SA response (Canet et al., 2012). HaRxL44, the nuclear-localized effector of *Hyaloperonospora arabidopsidis* (*Hpa*), interacts with MED19a, shifting the balance of defense transcription from the SA to the JA/ET-signaling pathway and increasing susceptibility to biotrophic pathogens by reducing SA-responsive gene expression (Caillaud et al., 2013). MED14, MED15, and MED16 have all been shown to be essential for the induction of systemic acquired resistance (Zhang et al., 2012, 2013; Wang et al., 2016). These results suggest that the Mediator complex acts as an integrative hub for regulation of specific signaling processes.

Here, we aimed to discover whether cotton cyclin-dependent kinase E (GhCDKE) plays a role in transcriptional response pathways, particularly those utilized in plant defenses against pathogen infection. To do this, we cloned GhCDKE, a subunit of the Mediator complex that interacts with CycC. We showed that GhCDKE is required for defense against *V. dahliae* infection, and we demonstrated that GhCDKE is required for a JA-mediated defense response after inoculation with *V. dahliae*. Our results indicated that GhCDKE is a key regulator of basal resistance against *V. dahliae*.

## Materials and Methods

### Plant Material

The seedlings of Zhongzhimian 2, a *V. dahliae*-tolerant breeding line of upland cotton, were cultivated under standard conditions of 26°C and a 16-h photoperiod. Tobacco (*Nicotiana benthamiana*) plants were grown in an incubator under controlled environmental conditions (22°C, 16 h photoperiod) as described previously (Talarczyk et al., 2002). *A. thaliana* seeds were germinated on Murashige and Skoog (MS) medium and cultured at 22°C under constant illumination, and 14-days-old seedlings were transferred to soil.

### RNA Extraction, RT-PCR, and qRT-PCR

Total RNA was isolated from cotton plants that were infected with Vd991 or exposed to 100 μM MeJA using an RNA extraction kit (Biomed, Enzo Life Sciences, Exeter, United Kingdom). The first strand of cDNA was synthesized from 2 μg of total RNA with a FASTQuant cDNA RT Kit (TIANGEN Biotech Co., Ltd) and used as template in quantitative reverse transcription polymerase chain reaction (qRT-PCR). The cotton Gh*UBQ* gene was used as an internal control. Primers were designed as shown in Supplementary Table S1. Reactions were prepared in 20-μL tubes using SYBR^®^-Premix Ex Taq (Tli RNaseH Plus; Takara, Shiga, Japan) and amplified on an ABI 7500 thermocycler (Applied Biosystems, Foster City, CA, United States). Expression was determined by the 2^-ΔΔCT^ method, and data were analyzed in Origin 8 (Liu et al., 2017).

### Disease Assays in Transgenic *Arabidopsis* Plants and Cotton

To obtain *GhCDKE*-overexpressing *Arabidopsis* plants, *GhCDKE* was amplified using the forward (5′-AAACTGCAGATGGGAGATGGCAATAACA-3′) and reverse (5′-TAGACTAGTCATGCGTCTCGATTTTTGC-3′) primers, which incorporated *Pat1* and *Spe1* cleavage sites, respectively. Next, the gene was cloned into vector Super-pCAMBIA1300. The plasmid was transformed into *Agrobacterium tumefaciens* strain GV3101. *Agrobacterium*-mediated transformation of *A. thaliana* was performed by floral dip method (Zhang et al., 2006).

The *V. dahliae* strain V991 was used for the inoculation assays. Fungal colonies were grown on potato-dextrose agar medium for 7 days and then the fungus was inoculated into Czapek medium at 25°C for 1 week at which time the spores were harvested. The spore suspension was adjusted to 10^6^ mL^-1^ with deionized water for the inoculation assays. Two-weeks-old cotton seedlings were removed from the soil and dip-infected into the spore suspension for 30 min. The seedlings were replanted in the soil and then harvested at 0, 0.5, and 12 h and 3, 5, and 7 days after inoculation. The transgenic *Arabidopsis* plants were inoculation with the same method. The disease ratio was calculated as the percentage of wilting plants to the total infected plants. The disease score criterion is occurrence of newly emerging leaves of infected *Arabidopsis* plants showing chlorosis phenotype (Gao X. et al., 2013).

### Yeast Two-Hybrid Assay

We used the pGADT7 vector containing the GAL4 AD region and the pGBKT7 vector containing GAL4 BD region for the yeast two-hybrid assay. The full-length coding sequence of GhCDKE was amplified with the listed primers (Supplementary Table S1). Enzyme-digested PCR products were cloned into the BD vector, and the coding region of the GhCDKE interacted with C-type cyclin (GhCycC) gene was cloned into the AD vector. About 500 ng of plasmid DNA of each construct was co-transformed into the yeast strain AH109. The yeast two-hybrid assays were based on Matchmaker GAL4 two-hybrid systems (Clontech). The presence of the transgenes was confirmed by growth on an SD/-Leu/-Trp plate. To assess protein interactions, the transformed yeasts were suspended in liquid SD/-Leu/-Trp to OD_600_ = 1.0, and 2 μL of suspended yeast was dripped onto an SD/-Ade/-His/-Leu/-Trp plate. Interactions were observed after 4 days of incubation at 30°C (Li et al., 2009).

### Luciferase Complementation Imaging Assay

LCI assay was conducted as described by Zhao et al. (2014). The vectors pCAMBIA1300-cLUC and pCAMBIA1300-Nluc were kindly provided by Prof. Zhizhong Gong (College of Biological Sciences, China Agricultural University). The full-length cDNA of GhCDKE was fused with NLUC in the pCAMBIA1300-Nluc vector, and that of GhCycC was fused with CLUC in the pCAMBIA1300-cLUC vector. The CLUC-GhERF6 and GhMLP28-NLUC constructs were used as positive controls (Yang et al., 2015). Next, the plasmid constructs were transformed into *A. tumefaciens* GV3101. Equal amounts (1:1) of *A. tumefaciens* cultures (OD_600_ = 1.0) containing the CLUC and NLUC constructs were mixed into the infiltration buffer (10 mM MES, pH 5.6; 200 mM acetosyringone; and 10 mM MgCl_2_) and then injected into *N. benthamiana* with a needleless syringe. Tobacco plants were kept in the dark for 24 h and then grown under normal conditions for 2 days. Infected leaves were sprayed with 1 mM luciferin (Sigma). A fluorescence image was obtained with a CCD camera (1300B; Roper).

### Virus-Induced Gene Silencing

The TRV vectors and *A. tumefaciens* GV3101 were prepared as described previously (Liu et al., 2017). At the same time, the *Cloroplastos alterados 1* (*CLA1*) gene was silenced at the initial establishment of the silent system and acted as a positive control (Supplementary Figure S3). The conserved regions of *GhCDKE* and *GhCLA1* (*CLA1*) were amplified from the cDNA of Zhongzhimian 2 with the primers shown in Supplementary Table S1 and then cloned into pTRV2 to generate pTRV2:GhCDKE and pTRV2:GhCLA1 (Gao et al., 2011). The constructs were then transformed into *A. tumefaciens* GV3101. The *A. tumefaciens* cultures were injected into the cotyledons of 2-weeks-old seedlings of Zhongzhimian 2 as described previously (Liu et al., 2017). Next, the seedlings were grown under normal conditions for 2 weeks. Next, expression of *GhCDKE* in infected cotton plants was detected by semi-qRT-PCR (Supplementary Figure S4). Subsequently, the GhCDKE-silenced cotton plants were challenged with *V. dahliae* by syringe inoculation as reported earlier (Liu et al., 2016).

### Promoter Isolation and Transient Expression

The promoter sequence of *GhPDF1.2* was obtained as previously reported (Yang et al., 2015). Primers were designed (Supplementary Table S1), and the gateway cloning system was used as described in the instruction manual (Invitrogen). The *GhPDF1.2* promoter sequence was cloned into vector pGWB435, which was kindly provided by Prof. Jinsong Zhang (Institute of Genetics and Developmental Biology, Chinese Academy of Sciences), to generate pGWB435-*GhPDF1.2_pro_:LUC*. Next, the construct was transformed into *A. tumefaciens* strain GV3101. The *A. tumefaciens* cultures were grown in LB medium containing 50 mg/mL rifampicin and 50 mg/mL spectinomycin. Next, *A. tumefaciens* cells harboring pGWB435-*GhPDF1.2_pro_:LUC* were treated with infiltration buffer for 3 h and then injected into the leaves of 4-week wild-type (WT) and *GhCDKE*-silenced cotton plants. Luminescence was measured 48 h after infiltration using the same procedure described for the luciferase complementation imaging (LCI) assay. Relative expression of *LUC* gene was also detected by qRT-PCR (Wang et al., 2015).

## Results

### Cloning and Sequence Analysis of *GhCDKE* and *GhCycC*

**FIGURE 1:**
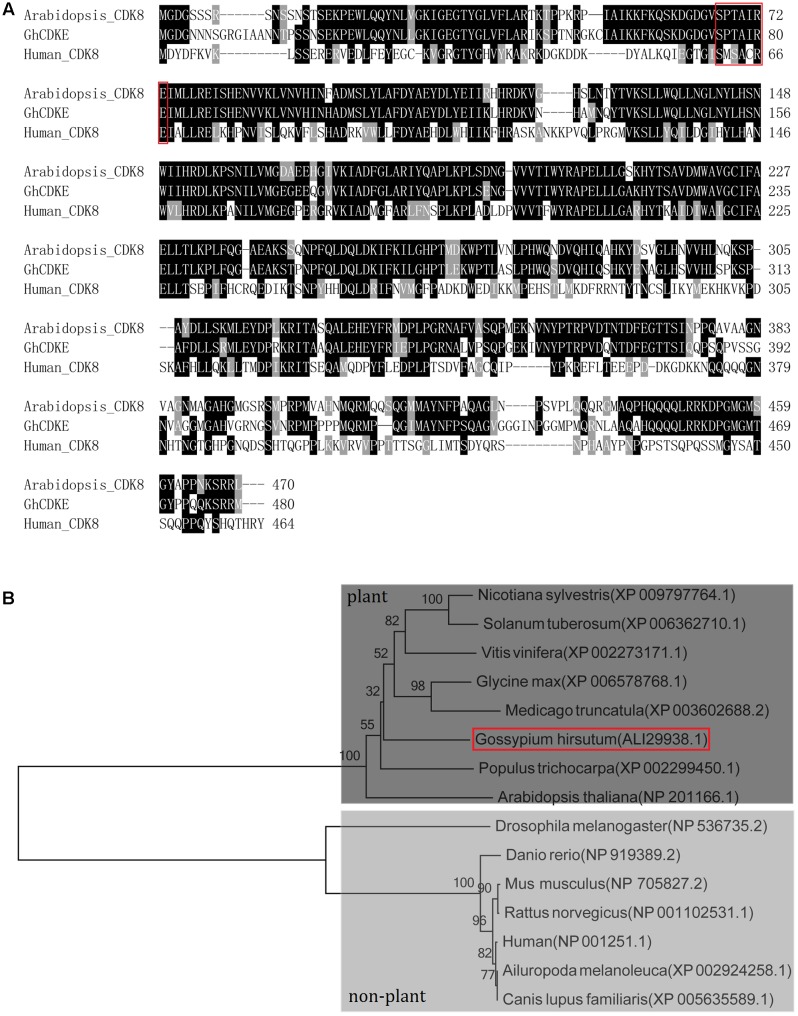
Amino acid sequence and phylogenetic analysis of GhCDKE. **(A)** Amino acid sequence alignment of GhCDKE homologs in different species. The sequences in the red rectangles are the putative cyclin binding motifs. Conserved residues are shaded in black and similar in gray. Amino acid sequence alignment was performed using Clutal *W*. Multiple alignment file was shaded using the BoxShade program. **(B)** Phylogenetic tree of GhCDKE homologs. The tree is divergent into two groups, the plant group and the non-plant group. The phylogenetic tree was performed using neighbor-joining method with MEGA 5.1.

To understand the molecular function of *GhCDKE*, we cloned this gene. The 1425-bp open reading frame (ORF) of *GhCDKE* was predicted to encode a protein of 474 amino acids with a predicted molecular mass of 53.42 kDa. CDK-related proteins in *Arabidopsis* were divided into seven conserved classes, CDKA–G, depending on the specific motif of their amino acid sequences, which are related to the binding of the corresponding cyclin (Vandepoele, 2002; Menges et al., 2005). Phylogenetic tree analysis showed that the protein belonged to the E-type CDKs (Supplementary Figure S1). Further, sequence alignment indicated that the protein contained the hallmark E-type CDK SPTAIRE motif involved in binding a specific cyclin, which corresponds to the SMSACRE motif in animal CDK8 amino acid sequences (**Figure 1A**). In addition, phylogenetic tree analysis showed that the E-type CDKs of many species have diverged into two groups: the plant group and the non-plant group (**Figure 1B**).

Various-type CDK with its characteristic hallmark binds the corresponding cyclin. In humans, cyclin C partners with CDK8 (Akoulitchev et al., 2000). In this study, we cloned the corresponding partner of GhCycC with 252 amino acids. Phylogenetic analysis showed that the partner was in the CycC group (Supplementary Figure S2).

### Interaction of GhCDKE With GhCycC

**FIGURE 2:**
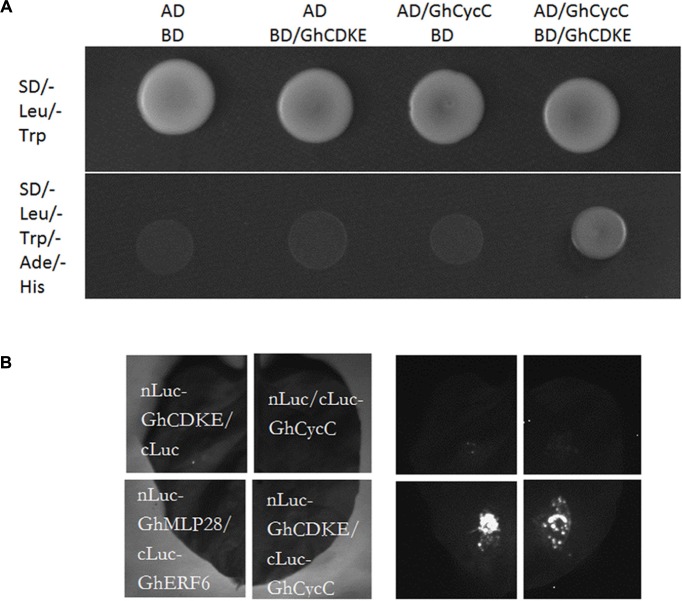
Interaction between GhCDKE and GhCycC. **(A)** Interaction between GhCDKE and GhCycC in yeast. GhCDKE was fused to the GAL4-binding domain (pGBKT7), and GhCycC was fused to the GAL4-activation domain (pGADT7). **(B)** Luciferase complementation imaging (LCI) assay of the interaction between GhCDKE and GhCycC in tobacco leaves. *Agrobacterium tumefaciens* GV3101 strains harboring constructs indicated on the left panel were transferred into tobacco leaves. LCI was performed 48 h after co-infiltration with the same amount of *A. tumefaciens* cells. Interaction between GhMLP28 and GhERF6 was used as the positive control.

Previous studies have shown that CDK8 and cyclin C interacted evolutionarily with each other in yeast, humans, and *Arabidopsis* (Tassan et al., 1995; Borggrefe et al., 2002; Andrau et al., 2006; Zhu et al., 2014). To elucidate whether this interaction occurred evolutionarily in cotton, we conducted a yeast two-hybrid assay. For this experiment, the GhCDKE protein was fused to the GAL4-DNA binding domain, and GhCycC was fused to the GAL4-activation domain (**Figure 2A**). As indicated in **Figure 2A**, GhCDKE interacted with GhCycC. In addition, an LCI assay was conducted to assess the interaction between GhCDKE and GhCycC in plant cells. The interaction between nLuc-GhMLP28 and cLuc-GhERF6 was used as the positive control, which was confirmed in a previous study (Yang et al., 2015). As shown in **Figure 2B**, the Luc signal was detectable only when cLuc-GhCycC/nLuc-GhCDKE or cLuc-GhERF6/nLuc-GhMLP28 was co-transformed into tobacco leaf cells. These results indicated that GhCDKE and GhCycC interact evolutionarily in cotton.

### Overexpression of GhCDKE Enhances Disease Tolerance of *Arabidopsis*

**FIGURE 3:**
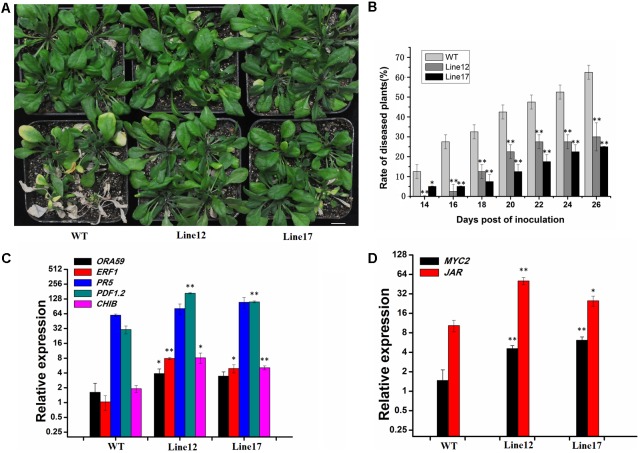
Disease symptoms of transgenic and wild-type plants. **(A)** Whole plant disease phenotype of wild-type and transgenic *Arabidopsis* infected with *V. dahliae*. Photos were taken 25 days post-inoculation. Bar = 10 mm. **(B)** Percentage of plants showing the *Verticillium* wilt phenotype at the indicated number of days after inoculation. The disease ratio was scored using 25 plants per line, and the assay was repeated three times with similar results. Expression (log2 scale) of JA-regulated pathogen-responsive genes **(C)** and wound-responsive genes **(D)** in wild-type and transgenic *Arabidopsis* (lines 12 and 17) plants after inoculation with *V. dahliae*. Data represent the mean ± *SD* of three independent biological repeats. Asterisks denote *t*-test significance compared with wild-type plants. ^∗^*p* < 0.05 and ^∗∗^*p* < 0.01. The data were analyzed by Student’s *t*-test using SPSS.

*Arabidopsis thaliana* Mediator complex subunits, such as MED18, MED25, and SFR6/MED16, are involved in regulating plant immunity (Çevik et al., 2012; Chen et al., 2012; Wathugala et al., 2012; Lai et al., 2014; Wang et al., 2016). Thus, we hypothesized that GhCDKE is likely involved in disease response. To test whether GhCDKE is required for resistance to *V. dahliae*, we ectopically overexpressed GhCDKE in *Arabidopsis*. Lines 12 and 17 showed the highest expression of *GhCDKE* (data not shown). Using a root-dipping inoculation assay, the newly emerging leaves showed chlorosis phenotype 14 days after inoculation. The chlorosis phenotype further progresses into the older leaves. Additionally, WT plants exhibited a more sensitive and severe wilting phenotype than transgenic plants did (**Figures 3A,B**, lines 12 and 17), indicating that GhCDKE limits pathogen growth and disease symptoms in plants infected with *Verticillium* wilt.

To study the role of GhCDKE in basal resistance to *V. dahliae*, we detected related gene expression involved in the JA pathway in WT and transgenic lines after inoculation. As shown in **Figure 3C**, expressions of JA-regulated pathogen-responsive genes (*ORA59*, *ERF1*, *PR5*, *PDF1.2*, and *CHIB*) were higher in lines 12 and 17 than WT plants in response to *V. dahliae* infection, especially the expressions of *PDF1.2* and *ERF1* which have been proved to function in resistance to *V. dahliae* or other fungi (Brown et al., 2003; Pré et al., 2008; Zhang et al., 2012; Xing et al., 2017). In addition, expressions of JA-regulated wound-responsive genes (*MYC2* and *JAR1*) were also activated by GhCDKE (**Figure 3D**) (Wassan, 2015). This result indicates that GhCDKE functions in the JA signaling pathway when plants are infected by pathogens.

### Knockdown of *GhCDKE* Expression Compromises Resistance of Cotton to *V. dahliae*

**FIGURE 4:**
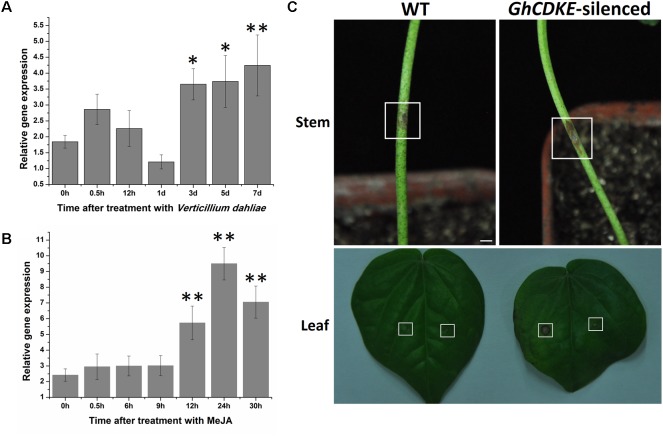
Enhanced cotton susceptibility to *V. dahliae* due to *GhCDKE* silencing. Expression profile of the *GhCDKE* gene in cotton after inoculation with *V. dahliae* **(A)** and treatment with MeJA **(B)**. Total RNA was extracted from 14-days-old cotton plants at the indicated times after treatment. Data are presented as mean ± *SD* of three biological replicates. ^∗^*p* < 0.05 and ^∗∗^*p* < 0.01 compared to 0 h by one-way ANOVA followed by LSD test. **(C)** Disease symptoms of wild-type and *GhCDKE*-silenced cotton inoculated with *V. dahliae*. The top panel shows stem symptoms. The bottom panel shows the leaf symptoms (left, wild-type; right, *GhCDKE*-silenced plants), and the rectangles mark the inoculation sites (left, inoculation with *V. dahliae*; right, inoculation with water as control). Bar = 3 mm.

To characterize the involvement of *GhCDKE* in cotton resistance to *V. dahliae*, we first determined its expression profile. As shown in **Figure 4A**, *GhCDKE* expression increased markedly at 0.5 h after inoculation of cotton seedlings with *V. dahliae* and reached the highest level at 7 days post-inoculation. To further investigate the involvement of *GhCDKE* in disease responses, we detected the expression of *GhCDKE* in cotton under treatment with methyl jasmonate (MeJA). As shown in **Figure 4B**, treatment of cotton seedlings with MeJA resulted in upregulation of *GhCDKE* expression at 12 h after treatment with expression peaking at 24 h. This result supports that *GhCDKE* is involved in resistance to *V. dahliae* infection.

Virus-induced gene silencing is widely employed to examine the various functions of genes (Liu et al., 2002; Fu et al., 2005; Gao et al., 2011; Gao X. et al., 2013; Yang et al., 2015). To further characterize the function of *GhCDKE* in cotton resistance to *V. dahliae*, we silenced *GhCDKE* by *Agrobacterium*-mediated VIGS. Next, we inoculated the WT and *GhCDKE*-silenced cotton lines with spore suspensions of *V. dahliae*. As shown in **Figure 4C**, leaves from *GhCDKE*-silenced cotton had larger disease lesions than those from WT cotton at 7 days after inoculation. Disease symptoms in stems were consistent with those in leaves. We conclude that *GhCDKE* reduces the severity of lesions caused by *V. dahliae* infection.

### GhCDKE Is an Important Regulator of JA Signal Pathway

**FIGURE 5:**
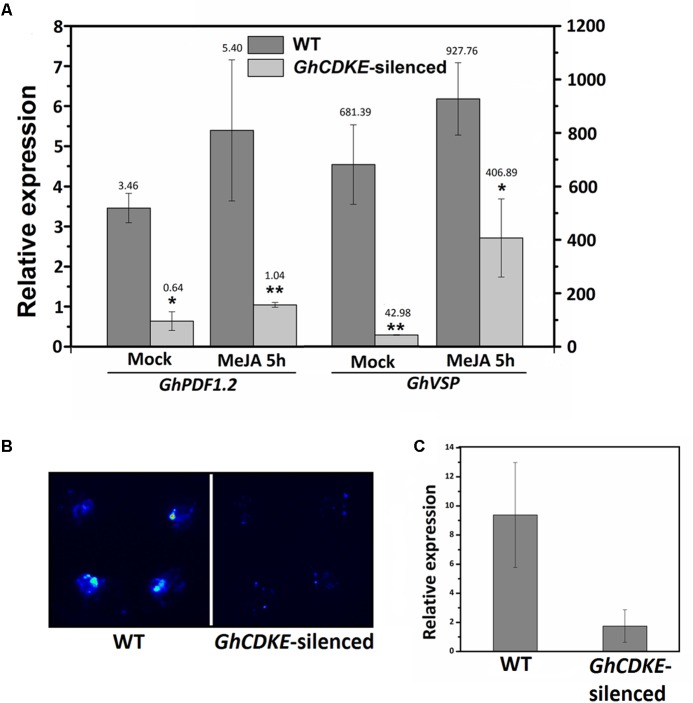
Expression assay of GhCDKE-dependent transcription of *GhPDF1.2* in cotton. **(A)** Quantitative RT-PCR analysis of *GhPDF1.2* and *GhVSP* in leaves of wild-type and *GhCDKE*-silenced cotton lines treated with or without 100 μM MeJA. The *GhUBQ7* gene was used as an endogenous reference gene. Data represent the mean ± *SD* of three biological replicates. ^∗^*p* < 0.05 and ^∗∗^*p* < 0.01 compared to 0 h by one-way ANOVA followed by LSD test. **(B)** Knockdown of *GhCDKE* reduces expression of *GhPDF1.2_pro_:LUC*. Four equal area sites were injected with the same amount of *Agrobacterium tumefaciens* cultures containing pGWB435-*GhPDF1.2_pro_:LUC* on every leaf. **(C)** Real-time polymerase chain reaction (RT-PCR) analyses of *LUC* transcripts from the samples in **(B)**.

Several subunits of the Mediator complex have been shown to be involved in hormone signaling regulation, especially the JA signaling pathway (Wathugala et al., 2012; Zhang et al., 2012; Yang et al., 2014). To determine the regulatory function of *GhCDKE* in the JA signal pathway, we evaluated the expression of JA-responsive marker genes (*GhVSP* and *GhPDF1.2*) in WT and *GhCDKE*-silenced cotton lines. As shown in **Figure 5A**, the level of *GhVSP* and *GhPDF1.2* transcripts in *GhCDKE*-silenced lines was lower than that in WT lines. The transcripts were induced by JA treatment, but fewer *GhVSP* and *GhPDF1.2* transcripts were found in *GhCDKE*-silenced lines. In addition, expression of *Luc* reporter gene driven by *GhPDF1.2* promoters was detected in WT and *GhCDKE*-silenced cotton plants. As shown in **Figure 5B**, promoter–reporter fusion assays revealed that *GhCDKE* knockdown inhibited expression *GhPDF1.2_pro_:Luc* (for luciferase). Consistent with this result, qRT-PCR assays showed that the expression of *Luc* gene driven by the *GhPDF1.2* promoter was markedly reduced in *GhCDKE*-silenced cotton lines (**Figure 5C**). These results suggested that GhCDKE functions as an important regulator in JA signal pathway.

### The Activated JA Response Caused by GhCDKE

**FIGURE 6:**
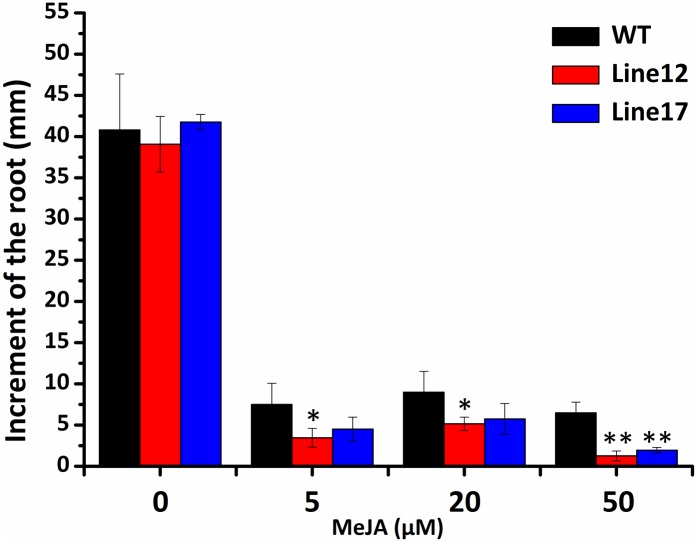
Root growth inhibition assay of 10-days-old seedlings from wild-type and transgenic *Arabidopsis* grown in Murashige and Skoog (MS) medium containing the indicated concentration of JA. Results are shown as the mean ± SD of measurements from 30 seedlings. ^∗^*p* < 0.05 and ^∗∗^*p* < 0.01 compared to 0 h by one-way ANOVA followed by LSD test.

Cotton cyclin-dependent kinase E regulated the expression of JA-responsive genes indicating its function in JA-signal pathway. To further characterize the role of GhCDKE in the JA signal pathway, we performed JA-induced root growth inhibition assays with overexpressed GhCDKE and WT lines. The assay showed that the transgenic lines were more sensitive than the WT lines to JA treatment in terms of root growth inhibition(**Figure 6**).

## Discussion

Upon perception by cotton cells, *V. dahliae* triggers genome-wide transcriptional reprogramming, such as with hormone pathway genes (Gao W. et al., 2013). Expression regulation of resistance-related genes plays a central role in plant response processes to various pathogens (Gao et al., 2011; Gao X. et al., 2013). Although much efforts in the field of disease resistance-related gene regulation are devoted to gene-specific transcription factors, we know relatively little about the function of the protein complexes that interact with RNA polymerase during gene transcription, such as the Mediator complex (Xu et al., 2004; Kim et al., 2008). Using molecular and genetic approaches, here we demonstrated the function of GhCDKE, a subunit of the Mediator complex. We found that first, GhCDKE physically interacted with its corresponding partner, GhCycC. Second, overexpression of GhCDKE in *Arabidopsis* conferred resistance to *V. dahliae* infection, and knockdown of it caused the opposite result. Third, GhCDKE was required for *V. dahliae*-induced JA-regulated pathogen-responsive gene expression.

The Mediator complex, an evolutionarily conserved multiprotein complex, functions in the transcription regulation of genes (Akoulitchev et al., 2000; Guglielmi et al., 2004; Kidd et al., 2011). It has been well studied in animal cells, especially the CDK8 subunit (Nemet et al., 2014). Mammalian CDK8 interacts with cyclin C to regulate the transcription of corresponding genes (Tassan et al., 1995; Akoulitchev et al., 2000; Schneider et al., 2011). The Srb10 also interacts with the Srb11 in yeast to function as a protein kinase (Borggrefe et al., 2002). Plant CDK8 was originally identified as HUA ENHANCER3 (HEN3) in *Arabidopsis*, which plays an important role in the specification of stamen and carpel identities (Wang and Chen, 2004). Subsequent reports have demonstrated that CDK8 interacts with CycCs to regulate plant immunity to fungal pathogens (Zhu et al., 2014). Here, we also showed that GhCDKE physically interacted with GhCycC suggesting a conserved configuration of plant Mediator kinase module and molecular mechanisms that underlie transcription regulation.

Studies have shown that the *PDF1.2* expression is reduced in *CDK8* mutants and regulated by WIN1/SHN1, a wax synthesis regulator that interacts with CDK8 (Zhu et al., 2014). PDF1.2 is involved in *V. dahliae* resistance (Yang et al., 2015). Thus, we used *PDF1.2* to test the transcriptional regulatory function of GhCDKE in cotton. Our results showed that the transcriptional activity of *PDF1.2* is dramatically decreased in GhCDKE-silenced cottons. Consistently, we observed that the expression level of *PDF1.2* was obviously increased in GhCDKE-overexpressed *Arabidopsis* plants. Based on our results, we conclude that *PDF1.2* may be a target of GhCDKE-mediated transcriptional regulation.

Previous studies have shown that JA plays a key role in plant–*V. dahliae* interactions (Derksen, 2011; Tan et al., 2015). In *Arabidopsis*, MED16 and MED25 are involved in the regulation of the JA/ET, SA, and ABA signal pathway (Chen et al., 2012; Zhang et al., 2012, 2013). Because the Mediator complex is conserved across various eukaryotes, we hypothesized that GhCDKE likely possesses similar functions in hormone signaling regulation. MED25 is involved in the JA signaling pathway including in the defense and wound response branches. Here, we showed that GhCDKE is also involved in JA signaling pathway (**Figures 3**, **5**, **6**). This result suggests that GhCDKE plays an essential role in relaying specific signals to RNAPII to regulate corresponding transcription. The mechanism by which GhCDKE recognizes different signals to activate corresponding gene transcription needs to be further studied.

*Arabidopsis thaliana* Mediator complex subunits, such as MED18, MED25, and SFR6/MED16, are involved in regulating plant immunity (Çevik et al., 2012; Chen et al., 2012; Wathugala et al., 2012; Lai et al., 2014; Wang et al., 2016). Thus, we hypothesized that GhCDKE is likely involved in disease response. Using the VIGS system, we found that silencing *GhCDKE* led to enhanced susceptibility to *V. dahliae*. Likewise, overexpression of GhCDKE in *Arabidopsis* increased resistance to *V. dahliae*. Taken together, these results indicated that GhCDKE-mediated defense may represent a broad-spectrum response in higher plants. Further studies regarding the mechanism of GhCDKE participation in defensive responses to various fungi need further study.

## Author Contributions

YH, FL, and XL conceived and designed the experiments. XL conducted most of the experiments, analyzed the data, and wrote the manuscript. NL, YS, and PW provided technical assistance to XL. NL and XG provided analysis tools. YP and DL contributed reagents and materials. All authors reviewed the manuscript.

## Conflict of Interest Statement

The authors declare that the research was conducted in the absence of any commercial or financial relationships that could be construed as a potential conflict of interest.
