# Supplementary material for: A Cotton Cyclin-Dependent Kinase E Confers Resistance to Verticillium dahliae Mediated by Jasmonate-Responsive Pathway
Source: Front Plant Sci. 2018 May 24;9:642. doi: 10.3389/fpls.2018.00642 (PMC5976743; doi:10.3389/fpls.2018.00642)
Supplement: Supplementary file 1 [file Data_Sheet_1.PDF]

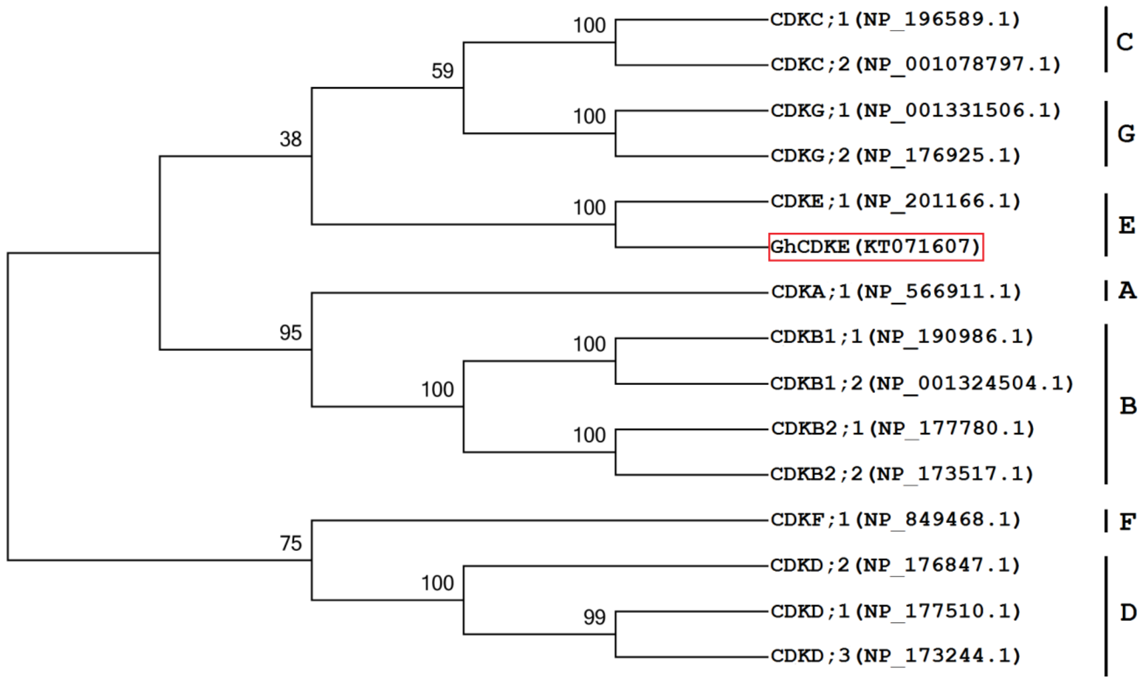

Supplemental Figure S1. Phylogenetic tree of seven groups of the CDK family. The evolutionary history was inferred using the Neighbor-Joining method. The percentage of replicate trees in which the associated taxa clustered together in the bootstrap test (1000 replicates) are shown above the branches. Evolutionary distances were computed using the Poisson correction method and are in units of the number of amino acid substitutions per site. Evolutionary analyses were conducted in MEGA5.1.

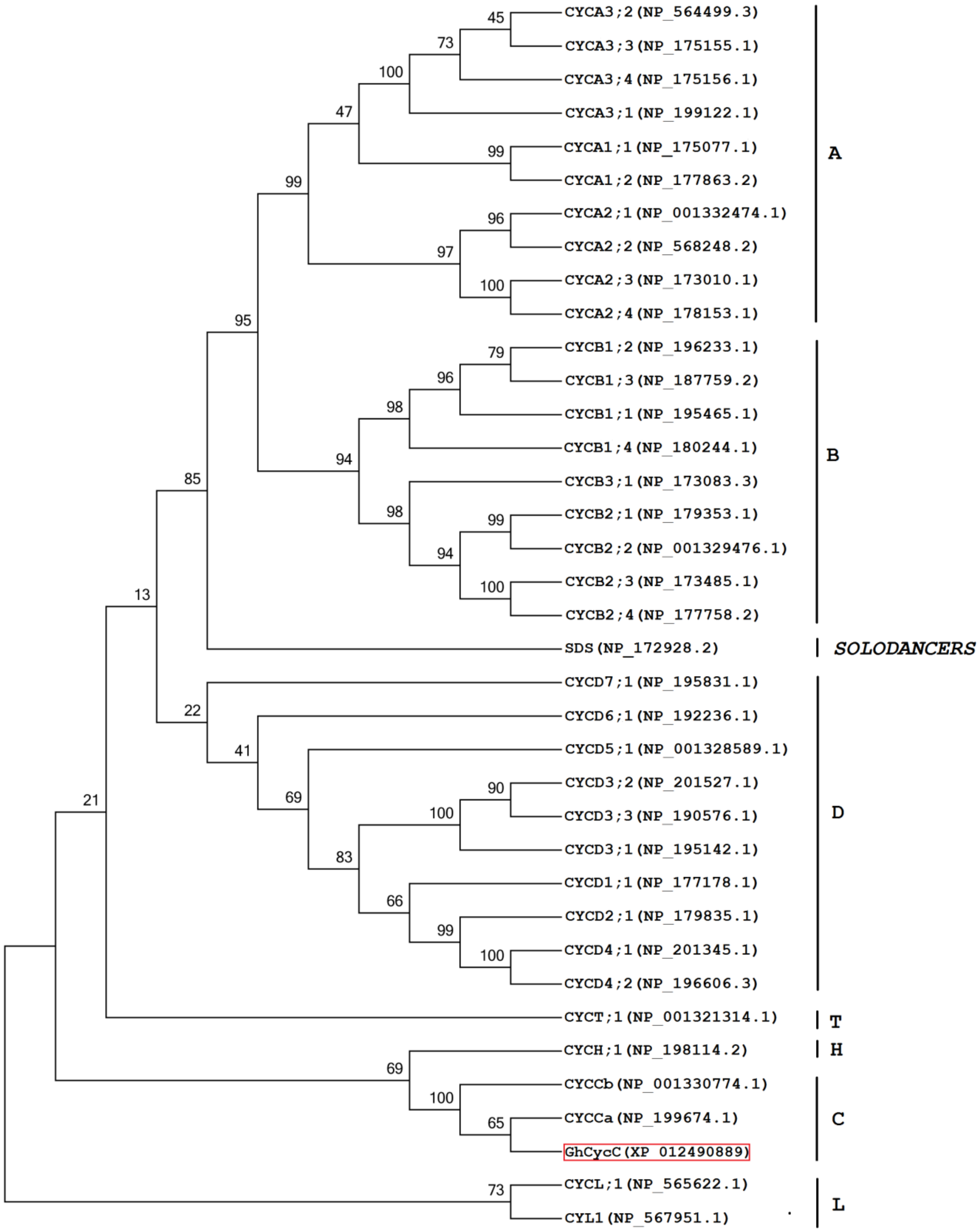

Supplemental Figure S2. Phylogenetic tree of eight groups of cyclins.

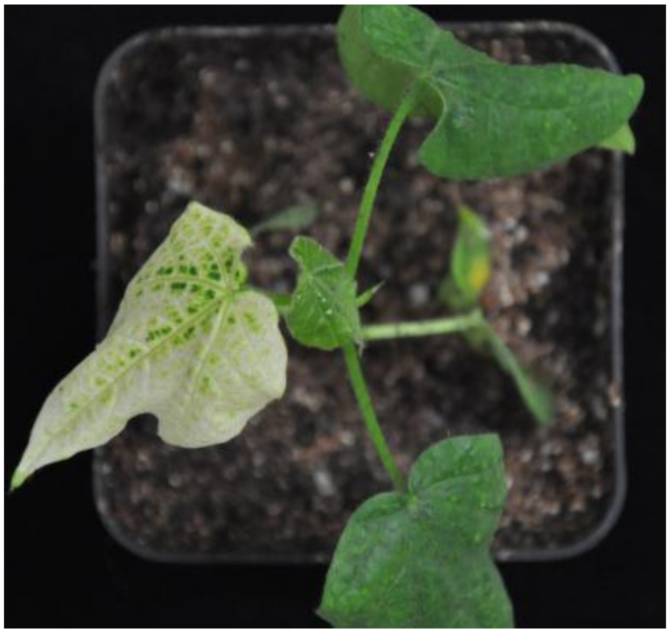

Supplemental Figure S3. VIGS-mediated silence of *GhCLA1* in cotton. The phenotype of cotton cotyledons inoculated with *Agrobacterium cultures* containing pTRV1 and pTRV2-GhCLA1.

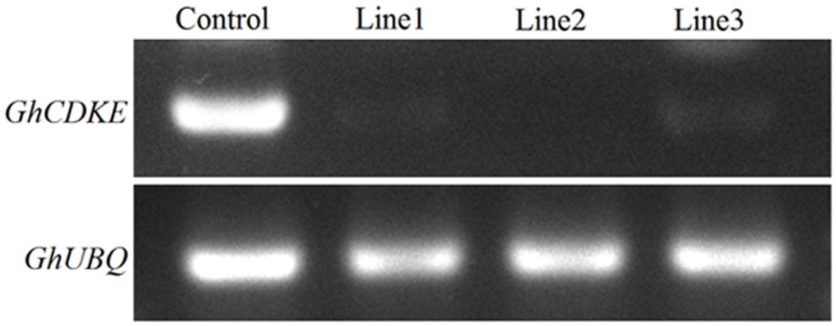

Supplemental Figure S4. Semi-quantitative real-time polymerase chain reaction (RT-PCR) analysis of the expression pattern of *GhCDKE* at the transcriptional level in wild-type and *GhCDKE*-silenced cottons. *GhUBQ7* was used as a reference gene. Expression of *GhCDKE* in the *GhCDKE*-silenced lines was dramatically lower than that in the wild-type cotton lines.

Supplenmental table S1. Primers used in this study.

Primer names and purpose	Primer sequences
GhCycC-F1,CLUC-GhCycC	5'-CCCGGTACCATGGCGGCCAATTTCTGGAC-3'
GhCycC-R1,CLUC- GhCycC	5'-GGGACTAGTTTAAGGCTTAAGAGCAAGTTTGAG-3'
GhCDKE-F1,NLUC-GhCDKE	5'-ATAGGATCCATGGGAGATGGCAATAACAAC-3'
GhCDKE-R1,NLUC-GhCDKE	5'-ATACTCGAGCATGCGTCTCGATTTTTGCTG-3'
GhCDKE-1300-F	5`-AAACTGCAGATGGGAGATGGCAATAACA-3`
GhCDKE-1300-R	5`-TAGACTAGTCATGCGTCTCGATTTTTGC-3`
qUBQ-F, qRT-PCR	5`-AGGCATTCCACCTGACCAAC-3`
qUBQ-R, qRT-PCR	5`- CAGCGAGCTTGACCTTCTTC-3`
qCDKE-F, qRT-PCR	5`-CTACTCGTCCGGTGGACCAG-3`
qCDKE-R, qRT-PCR	5`-TGATGAGCTTGGGCAGCAAG -3`
qCHIB-F, qRT-PCR	5`-CAATGTGGTCGCCAAGCTGGAGG-3`
qCHIB-R, qRT-PCR	5`-TGAAATGATGCCCGAAAGATCGCC-3`
qPR5-F, qRT-PCR	5`-TGTTCATCACAAGCGGCATTGCTG-3`
qPR5-R, qRT-PCR	5`-GTGAGCTGTCGGGAAGCACCTG-3`
AtEF-1-α-F, qRT-PCR	5'-CCTGGATTGCCACACC-3'
AtEF-1-α-R, qRT-PCR	5'-AGTCTGCCTCATGTCC-3'
qPDF1.2-F, qRT-PCR	5`-GTGGAAGCACAGAAGTTGTGC-3`
qPDF1.2-R, qRT-PCR	5`-ACACTTGTGTGCTGGGAAGAC-3`
qERF1- F, qRT-PCR	5`-GGAGAGAGTTCAAGAGTCGCTT-3`
qERF1- R, qRT-PCR	5`-AGCTCCTCAAGGTACTGTTCTC -3`
qGhVSP-F, qRT-PCR	5`-TTGTTCGATGAGTGGGTAATGG-3`
qGhVSP-R, qRT-PCR	5`-CCTGAATACTTTGATGGTTCCTTG-3`
qORA59 F, qRT-PCR	5`-GAGGAGGAGGAGAAGTCATACA-3`
qORA59 R, qRT-PCR	5`-AAAGCCGCCTGATCATAAGCGA-3`
CDKE-F3, pTRV-GhCDKE	5`-CGACGACAAGACCGTGACCATGGCACCTGAATTGCTTCTTGG-3`
CDKE-R3, pTRV-GhCDKE	5`-GAGGAGAAGAGCCGTCATTATGCAGCAGTTATACGCTTAC-3`
GhCLA1-F, pTRV-GhCLA1	5`-CGACGACAAGACCGTGACCATGCACAACATCGATGATTTAG-3`
GhCLA1-R, pTRV-GhCLA1	5`-GAGGAGAAGAGCCGTCATTAGCATGAATGATGAGTAGATTGCAC-3`
GhPDF1.2$_{Pro}$-F, pGWB435-GhPDF1.2$_{Pro}$	5'-ATTCCTCGAAAAATAATAATCTAGAAAGA-3'
GhPDF1.2$_{Pro}$-R, pGWB435-GhPDF1.2$_{Pro}$	5'-TAAATATTATTATAACCGAGCGAATGATTGAAA-3`
CDKE-BD-F, pGBKT7-GhCDKE	5'-GCGGAATTCATGGGAGATGGCAATAACA-3'
CDKE-BD-R, pGBKT7-GhCDKE	5'-GCGCTGCAGCATGCGTCTCGATTTTTGC-3'
Cyc7-AD-F, pGADT7-GhCycC	5'-GCGGAATTCATGGCGGCCAATTTCTGGACTTC-3'
Cyc7-AD-R, pGADT7-GhCycC	5'-GCGCTGCAGCAGGCTTAAGAGCAAGTTTGAGG-3'
